# The Risk G Allele of the Single-Nucleotide Polymorphism rs928413 Creates a CREB1-Binding Site That Activates *IL33* Promoter in Lung Epithelial Cells

**DOI:** 10.3390/ijms19102911

**Published:** 2018-09-25

**Authors:** Alisa M. Gorbacheva, Kirill V. Korneev, Dmitry V. Kuprash, Nikita A. Mitkin

**Affiliations:** 1 Laboratory of Intracellular Signaling in Health and Disease, Engelhardt Institute of Molecular Biology, Russian Academy of Sciences, Moscow 119991, Russia; alisamur93@mail.ru (A.M.G.); kirkorneev@yandex.ru (K.V.K.); kuprash@gmail.com (D.V.K.); 2 Biological Faculty, Lomonosov Moscow State University, Moscow 119234, Russia

**Keywords:** asthma, lung epithelium, inflammation, p38 MAPK pathway

## Abstract

Cytokine interleukin 33 (IL-33) is constitutively expressed by epithelial barrier cells, and promotes the development of humoral immune responses. Along with other proinflammatory mediators released by the epithelium of airways and lungs, it plays an important role in a number of respiratory pathologies. In particular, IL-33 significantly contributes to pathogenesis of allergy and asthma; genetic variations in the *IL33* locus are associated with increased susceptibility to asthma. Large-scale genome-wide association studies have identified minor “G” allele of the single-nucleotide polymorphism rs928413, located in the *IL33* promoter area, as a susceptible variant for early childhood and atopic asthma development. Here, we demonstrate that the rs928413(G) allele creates a binding site for the cAMP response element-binding protein 1 (CREB1) transcription factor. In a pulmonary epithelial cell line, activation of CREB1, presumably via the p38 mitogen-activated protein kinases (MAPK) cascade, activates the *IL33* promoter containing the rs928413(G) allele specifically and in a CREB1-dependent manner. This mechanism may explain the negative effect of the rs928413 minor “G” allele on asthma development.

## 1. Introduction

Interleukin 33 (IL-33), a member of the interleukin 1 (IL-1) cytokine family, is expressed by endothelial, epithelial, and fibroblast-like cells in barrier tissues and in the sites of inflammation [1,2]. This cytokine also acts as alarmin that is released upon necrotic cell death or stress mediated by infectious agents [3]. Secreted IL-33 binds to heterodimer receptor complex that is located on the surface of multiple myeloid and lymphoid cell types, and is formed by specific receptor ST2 (interleukin 1 receptor-like 1, IL1RL1) and co-receptor interleukin-1 receptor accessory protein (IL-1RAcP). Receptor binding induces activation of NF-κB and mitogen-activated protein kinase (MAPK) signaling pathways [1,4]. IL-33 signaling is associated with induced proliferation of basophils, eosinophils, mast cells, natural killer T (NKT) and type 2 helper T cells, their directed migration, and elevated production of type 2 immune mediators, interleukin 4 (IL-4), interleukin 5 (IL-5) and interleukin 13 (IL-13) [5,6]. The IL-33/ST2 pathway is especially accompanied by strong activation of type 2 innate lymphoid cells, which appear to be an important source of IL-5 and IL-13, which are essential for asthma pathogenesis [7].

Numerous studies have demonstrated the particular importance of IL-33 in respiratory and allergic diseases [8,9]. IL-33 level correlates with clinical symptoms in asthmatic patients, with elevated IL-33 protein observed in lung epithelium [9], airway smooth muscle [8], and epithelial cells [10], serum [11] and bronchoalveolar lavage fluid [12]. Elevated IL-33 stimulates systemic T-helper 2 (Th2) type of inflammation and contributes to allergen-induced airway inflammation and hyper-responsiveness, aggravating asthma symptoms [10].

Large-scale genome-wide association studies demonstrated that *IL33* locus includes several single-nucleotide polymorphisms (SNPs) associated with asthma development in different populations [13,14,15,16]. One of them, rs928413 (A/G), is located in the 5′ upstream region of *IL33* gene, and its minor “G” allele was identified as a susceptible variant for early childhood asthma [16] and atopic asthma [17] development. Of note, both homozygous and heterozygous carriers of the rs928413(G) risk allele demonstrated exacerbation of clinically important asthma symptoms [17]. The rs928413(G) allele also demonstrated an association with increased risk of other childhood atopic phenotypes such as hay fever [18], also pointing at possible involvement of this non-coding polymorphism in the development of allergic inflammation.

Based on the available data, we hypothesized that the rs928413(G) allele may be mechanistically linked to asthma susceptibility via transcriptional activation of the *IL33* gene. In this paper, we demonstrate that the presence of rs928413(G) allele in the *IL33* promoter indeed results in its increased activity in a human lung carcinoma cell line due to binding of the cAMP (cyclic adenosine monophosphate) response element binding protein 1 (CREB1) transcription factor. Our results offer a tentative explanation for the negative effect of rs928413 on asthma development.

## 2. Results

### 2.1. Presence of “G” Allele of rs928413 Is Associated with Increased IL33 Promoter Activity

**Figure 1:**
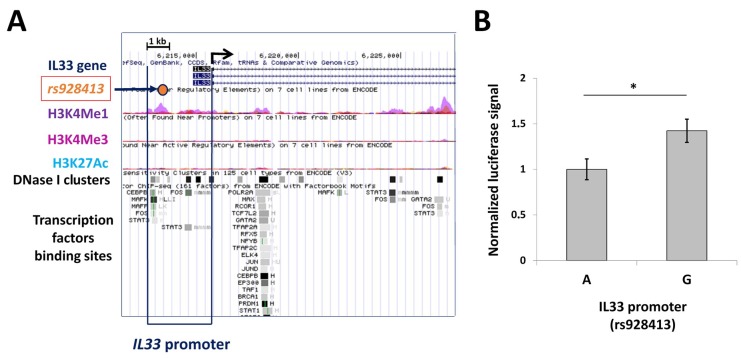
Minor G allele of rs928413 located in human *IL33* promoter increases its activity in a luciferase reporter system. (**A**) Illustration of the 5′ area of human *IL33* gene (UCSC Genome Browser, GRCh37/hg19 assembly) with selected maps of histone modifications, DNase I hypersensitivity clusters and predicted transcription factors binding sites. H3K4Me1: the track indicating areas of monomethylation of Histone H3 at lysine 4; H3K4Me3: the track indicating areas of trimethylation of Histone H3 at lysine 4; H3K27Ac: the track indicating areas of acetylation of Histone H3 at lysine 27; DNase I cluster: the track indicating deoxyribonuclease I hypersensitivity clusters. Horizontal arrow points to Rs928413 location. Angled arrow indicates transcription start site. (**B**) Rs928413(G) allele of the human *IL33* promoter has elevated activity in lung cancer cells. A: *IL33* promoter containing Rs928413(A) allele; G: *IL33* promoter containing Rs928413(G) allele. The experiment was performed five times. * *p* < 0.05.

The regulation of human *IL33* transcription in different cell types is rather complex, and the available data is rather limited [19,20]. Bioinformatic data on the human *IL33* gene available through UCSC Genome Browser (Available online: http://genome.ucsc.edu) is consistent with the activity of the most distant promoter in a broad range of tissues, with epigenetic features which are characteristic of regulatory elements such as DNase-I hypersensitivity sites, major histone modifications (H3K4me3: trimethylation of Histone H3 at lysine 4; H3K4me1: monomethylation of Histone H3 at lysine 4; H3K27ac: acetylation of Histone H3 at lysine 27), and the presence of transcription factors (TF) binding sites spanning the 2,5 kb region upstream of the transcription start site (TSS) (Figure 1A). Single-nucleotide polymorphism rs928413 is located in this area, and its minor G allele was shown to be associated with increased risk of asthma development [16,17]. To evaluate the effect of rs928413 nucleotide variations on *IL33* promoter activity, we generated luciferase reporter constructs containing *IL33* promoter variants in the pGL3-Basic vector. These constructs were tested in a NCIH-196 human lung carcinoma cell line, which is characterized by constitutive *IL33* expression, according to Broad-Novartis Cancer Cell Line Encyclopedia [21]. The reporter construct containing the rs928413(G) allele was significantly more active in this assay (Figure 1B).

### 2.2. Risk Allele of rs928413 in IL33 Promoter Creates CREB1-Binding Site

The difference in the activity between *IL33* promoter variants containing alternative rs928413 alleles is likely associated with the emergence or disappearance of specific transcription factor binding sites (TFBS). We used the PERFECTOS-APE software (Available online: http://opera.autosome.ru/perfectosape, Moscow, Russia) [22] with TFBS models from HOCOMOCO (Available online: http://hocomoco11.autosome.ru) and JASPAR databases (Available online: http://jaspar.genereg.net) to generate a list of candidate transcription factors with the best scores, which included CREB1 (cAMP response element-binding protein 1), FOXF2 (forkhead box protein F2), HNF1A (hepatocyte nuclear factor 1 homeobox A), and E4F1 (E4F transcription factor 1). Of these four, three proteins had no apparent connection to the pathogenesis of asthma: Foxf2 has been shown to play a role in embryogenesis [23], in particular in the development of gut [24] and secondary palate [25], HNF1A is involved in control of liver-specific gene expression [26] and in glucose metabolism [27], and E4F1 is necessary for the proliferation and survival of embryonic, stem, and cancer cells [28,29,30]. On the other hand, phosphorylated CREB1 is known as an important activator of various genes encoding chemokines and pro-inflammatory cytokines that are biologically relevant to asthma [31]. Importantly, CREB has been implicated in asthmatic pathogenesis [32,33,34]. However, the physiological significance of CREB phosphorylation in lung epithelium has not been sufficiently studied, and functional mechanisms mediating the association between CREB phosphorylation and asthma remain unclear [35]. We hypothesized that the binding site for CREB1 created by the risk rs928413(G) allele resulting in enhanced *IL33* transcription may provide a mechanistical explanation for this association.

**Figure 2:**
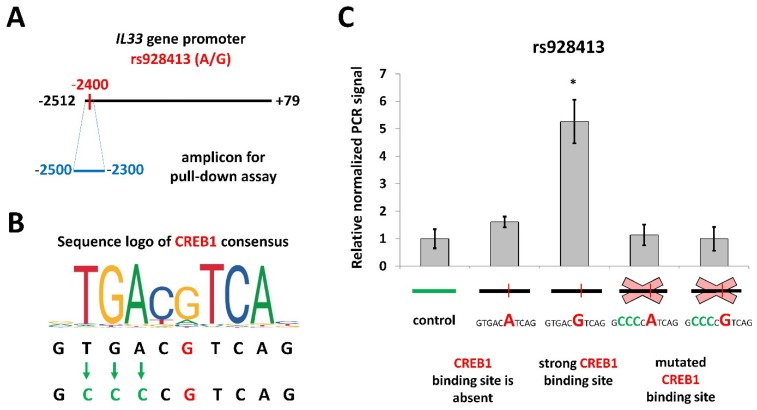
Rs928413(G) is associated with CREB1 binding. (**A**) Schematic illustration of rs928413 location in *IL33* promoter and the amplicon used for the pull-down assay. (**B**) Position weight matrix of CREB1-binding site and schematic illustration of its mutagenesis. Green arrows indicate nucleotides changes. (**C**) Efficiency of CREB1 binding to different variants of *IL33* promoter (rs928413 variants shown in red color) as estimated by pull-down assay using nuclear extracts from NCIH-196 cells. Data was normalized to control amplicon after subtraction of the background signal (see Section 4). The result of five independent experiments is represented. * *p* < 0.01.

We used a pull-down assay with nuclear extracts from NCIH-196 cell line to investigate the influence of rs928413 on CREB1 binding to the *IL33* promoter. We created four variants of the *IL33* gene promoter region (−2500/−2300), containing either common “A” or risk “G” rs928413 allele, and either the original or mutated CREB1 site (Figure 2). The probe containing the rs928413(G) allele demonstrated high precipitation efficiency with anti-CREB1 antibodies, while the level of precipitation of the fragment containing the “A” allele did not significantly differ from that of control probes. Thus, a CREB1-binding site is indeed present in the human *IL33* promoter containing risk rs928413(G) allele.

### 2.3. CREB1 Activation Is Associated with Elevated IL33 Promoter Activity

Activation of CREB by phosphorylation at serine 133 is promoted by various type of kinases, including mitogen-activated protein kinases (MAPKs) [31,36]; p38 MAPK is hyper-activated in the cells of patients with severe asthma [37,38]. The activity of p38 MAP kinases is, in turn, induced by inflammatory mediators [39] including TNF (tumor necrosis factor), which is also involved in asthma pathogenesis [40]. In particular, CREB phosphorylation through p38 MAP kinases after TNF stimulation was demonstrated in human airway epithelial cells [41] and in primary lung fibroblasts in asthma patients [35]. Therefore, we tested the effects of TNF in our model system.

**Figure 3:**
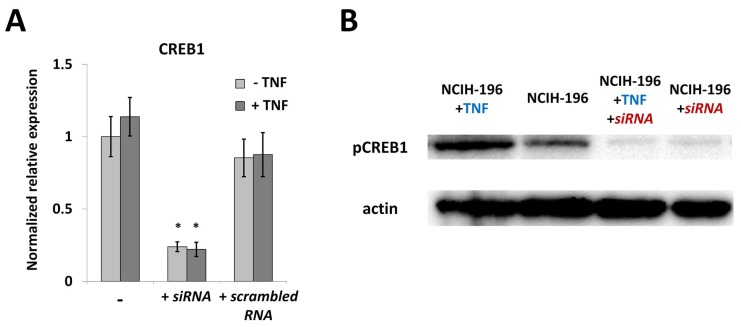
TNF-induced activation promotes phosphorylation of CREB1 in lung cancer cells. Activation by TNF (tumor necrosis factor) (100 ng/mL, 24 h) does not influence the level of *CREB1* expression (**A**) but results in an increase in phosphorylated CREB1 protein (**B**). In (**A**), NCIH-196 cells were: “-”, without addition of any siRNA; “+ *siRNA*”, transfected with CREB1-specific siRNA; “+ *scrambled RNA*” transfected with scrambled siRNA. In (**B**), NCIH-196 cells were: “NCIH-196 + TNF”, incubated with TNF; “NCIH-196”, without TNF or siRNA;”"NCIH-196 + TNF + *siRNA*”, transfected with siRNA and incubated with TNF; “NCIH-196 + *siRNA*”, transfected with siRNA only. Real-time PCR data is determined using the ΔΔ*C*t approach, normalized to β-actin and represented as Mean ± SEM (five replicate experiments). * *p* < 0.01. Western blot data is a representative image of three independent experiments.

As expected, treatment with TNF for 24 h did not affect the level of *CREB1* mRNA in NCIH-196 cells (Figure 3A), while phosphorylated CREB1 (p-CREB1) was significantly activated by the cytokine (Figure 3B). Transfection with specific siRNA resulted in a strong decrease in both *CREB1* mRNA and p-CREB1 levels, regardless of the TNF presence (Figure 3A,B).

**Figure 4:**
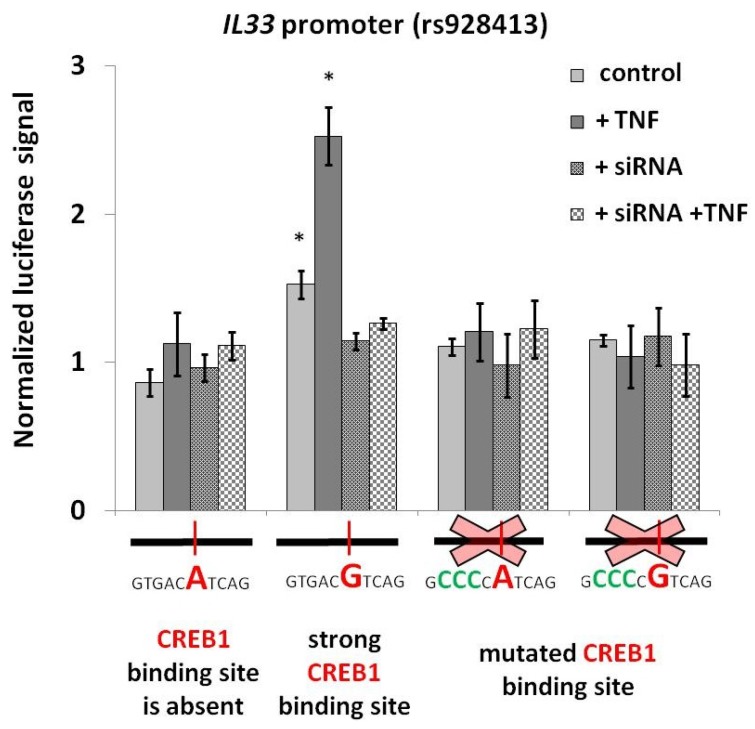
The rs928413(G) allele mediates CREB1-dependent *IL33* promoter stimulation of lung cancer cells by TNF. Reporter constructs contained Firefly luciferase gene under the control of indicated versions of the *IL33* promoter. siRNA for the *CREB1* knockdown was electroporated 24 h prior to electroporation of the reporter. TNF (100 ng/mL) was added at the time of both electroporations. The data shown (mean ± SEM) was obtained in five independent experiments and normalized to Renilla luciferase activity. * *p* < 0.01.

In order to determine the role of CREB1 in *IL33* promoter activity, we combined the panel of reporter vectors bearing rs928413 allelic variants and CREB1-binding site mutations with TNF activation and siRNA-mediated *CREB1* knockdown. As shown on Figure 4, TNF activation selectively and specifically increased the activity of the *IL33* promoter construct containing rs928413(G) allele and functional CREB1-binding site. Both the presence of rs928413(A) allele and the CREB1 site mutation made the reporter unresponsive to TNF. Activation was also completely abrogated by the addition of *CREB1*-specific siRNA. These results indicate that the potentially negative role of the minor rs928413(G) allele in asthma may be mechanistically explained by *IL33* promoter stimulation with CREB1 transcription factor, that can be further activated by TNF.

## 3. Discussion

The epithelium of airways and lungs is one of the most important physical barriers between external environment and the human organism. Due to permanent interaction with pathogenic microorganisms and inhaled antigens, lung epithelial cells (ECs) act as mediators of the host’s defense [42], and often become a key participant in the development of allergic inflammation and asthma pathogenesis [43]. Lung ECs express a wide repertoire of pattern-recognition receptors and protease activated receptors that can be stimulated directly by pathogens and by allergens during the sensitization phase of asthma [44,45,46,47]. Activated epithelial cells release chemokines [48,49], danger-associated molecular patterns (DAMPs) [50,51] and cytokines [48,52,53] which attract various immune cells to the airways and lungs, and modulate their response [7,54,55]. IL-33 is considered to be one of the crucial epithelial-derived cytokines involved in allergic inflammation and asthma development. It stimulates airway hyper-responsiveness [56] and induction of Th2-type adaptive immunity [57], expansion of type 2 innate lymphoid cells [58], mobilization of eosinophils [59], mast cells and basophils activation [60,61], and an increase in IgE production by B-cells [62]. IL-33 blockade contributes to the alleviation of allergic rhinitis symptoms [63], and suppresses the development of asthma in mouse models [64,65,66]. Accordingly, elevated expression of *IL33* in the airway and lung epithelium is supposed to be associated with the maintenance of mucosal inflammatory conditions and the aggravation of pathological changes during asthma.

In the present work, we characterized a possible functional role of SNP rs928413 located in the distal promoter area of the *IL33* gene. We demonstrated that the risk rs928413(G) allele is associated with the presence of the CREB1 binding site and increased *IL33* promoter activity, which can be enhanced by TNF, presumably via p38 MAPK cascade. As already noted, increased activation of this pathway was observed in the asthmatic airways; the extent of the activation correlated with the severity of the disease [37,38]. Furthermore, p38 kinase appears to play an important role in asthma exacerbation by controlling production of proinflammatory cytokines and chemokines by epithelial cells [67]. Elevated CREB1 activity has been linked by many studies to the regulation of proinflammatory cytokines production [68,69] and to the pathogenesis of respiratory diseases such as chronic obstructive pulmonary disease (COPD) [70]. In the case of asthma, the level of CREB1 phosphorylation in bronchial epithelium of asthmatic patients correlated with the inflammatory status, and consequently, could be used as a prognostic marker [34]. CREB1 binding to a site in the *IL33* promoter created by the rs928413(G) allele is consistent with the observation of more pronounced clinical asthma symptoms in patients carrying both homozygous and heterozygous rs928413(G) variants [17].

In conclusion, our data suggest that a mechanism that may explain the observed association of rs928413 polymorphism with asthma susceptibility. Elevated production of IL-33 by damaged or stressed cells of the barrier epithelium could promote airway hyper-responsiveness and development of inflammatory response during the initiation and persistence of asthma. The proposed mechanism corroborates the critical role of epithelium in asthmatic pathogenesis reported in an increasing number of studies. In the future, it is important to assess the correlation between CREB1 binding to the rs928413(G) allele with IL-33 production in primary cells, and with disease symptoms in asthmatic patients.

## 4. Materials and Methods

### 4.1. Cell Lines

A NCIH-196 human lung carcinoma cell line was obtained from American Type Culture Collection (ATCC, Manassas, VA, USA). Cells were cultured in RPMI-1640 medium (Life Technologies, Carlsbad, CA, USA) supplemented with 10% fetal bovine serum (Thermo Scientific, Waltham, MA, USA). Preliminary experiments with various concentrations of recombinant human TNF (tumor necrosis factor) (kindly provided by Daniela N. Männel, Institute of Immunology, University of Regensburg, Regensburg, Germany) (1, 10, 100 and 300 ng/mL) for 24 and 48 h demonstrated that 100 ng/mL TNF for 24 h yielded the strongest increase in *IL33* expression, as measured by real-time PCR. Therefore, this treatment regiment was used in further experiments.

### 4.2. Ethical Approval

The Scientific Council of the Engelhardt Institute of Molecular Biology declared no ethical approval requirements for experiments performed in this study, because only commercially available cell lines were used.

### 4.3. Luciferase Reporter Constructss

The human *IL33* promoter region containing nucleotides −2512 to +79 bp from the transcription start site (TSS) was amplified by PCR using genomic DNA isolated from NCIH-196 cells and specific oligonucleotide primers containing HindIII/NcoI restriction sites. *IL33* promoter variants containing the minor (G) allele of rs928413 and mutations of the CREB1 binding site were amplified by overlap PCR, and verified by Sanger sequencing. Variants of *IL33* promoter were cloned into pGL3-basic luciferase reporter vector (Promega, Madison, WI, USA) using HindIII and NcoI restriction sites. The sequences of the primers are indicated in Table S1.

### 4.4. NCIH-196 Transfection and Luciferase Reporter Assay

Сells were transfected with 1.8 μg of plasmid DNA and 0.2 μg of pRL-CMV Renilla luciferase control vector using X-treme Gene HP DNA Transfection Reagent (Roche, Basel, Switzerland) according to manufacturer’s protocol. Firefly luciferase activity was measured as described [71], and normalized to the activity of Renilla luciferase in order to account for the variations in cell transfection and lysis efficiencies.

### 4.5. Pull-Down Assay

Two hundred bp fragments of the *IL33* promoter (−2500 to −2300 from the TSS) containing rs928413 were amplified by PCR using DNA templates containing combinations of the A and G rs928413 alleles with either intact or mutant CREB1 binding site. An irrelevant DNA fragment that did not include any predicted CREB1-binding sites was used as a negative control. All PCR products were verified by sequencing; sequences of PCR primers are represented in Table S1. Isolation of nuclear extracts, formation and immunoprecipitation of DNA-protein complexes, and the evaluation of bound DNA probes by RT-PCR were performed as described [72]. Rabbit polyclonal anti-CREB1 antibody (ab31387, Abcam, Cambridge, UK) was precipitated with pre-blocked magnetic beads (Thermo Scientific, Waltham, MA, USA). The background signal was determined using a negative control DNA fragment and binding reactions without nuclear extracts, without antibodies and with rabbit IgG isotype control. All data was normalized to the signal obtained with negative control DNA probe.

### 4.6. CREB1 Knockdown Using siRNA

Commercially synthesized single-stranded RNAs (Syntol, Moscow, Russia) were annealed as previously described [73]. NCIH-196 cells were transfected with siRNA duplexes (500 pmol of per 5 million cells) 48 h before transfection with the luciferase constructs and a further 200 pmol to extend the silencing effect. The measurement of *CREB1* mRNA level by RT-PCR was performed as described [74]. Of three published pairs of siRNAs against CREB1 [75], one mediated a significant decrease in *CREB1* mRNA level. The sequences of the siRNA and the *CREB1*-specific PCR primers are represented in Table S1.

### 4.7. Western Blot Analysis

Protocols of total cell lysates preparation, electrophoresis, and transfer to the nitrocellulose membrane were previously described [76]. Membranes were blocked with 5% non-fat dry milk, and incubated with anti-CREB1 (phospho S133) antibodies (ab32096, Abcam, Cambridge, UK) at 1:2000 dilution and anti-β-actin antibodies (ab8229, Abcam, Cambridge, UK) at 1:3000 dilution as a loading control. The bands were visualized using SuperSignal West Dura Extended Duration Substrate (Thermo Scientific, Waltham, MA, USA) and ChemiDoc XRS + system (BioRad, Hercules, CA, USA).

### 4.8. Statistical Analysis

We used Microsoft Excel for statistical analyses. Statistical significance was determined using two-tailed unpaired Student’s *t*-test. Data were represented as mean ± SEM.

## Supplementary Materials

Supplementary materials can be found at http://www.mdpi.com/1422-0067/19/10/2911/s1.
