# Supplementary material for: The Risk G Allele of the Single-Nucleotide Polymorphism rs928413 Creates a CREB1-Binding Site That Activates IL33 Promoter in Lung Epithelial Cells
Source: Int J Mol Sci. 2018 Sep 25;19(10):2911. doi: 10.3390/ijms19102911 (PMC6212888; doi:10.3390/ijms19102911)
Supplement: Supplementary file 1 [file ijms-19-02911-s001.pdf]

Supplementary materials

The Risk G Allele of the Single-Nucleotide Polymorphism rs928413 Creates a CREB1-Binding Site that Activates *IL33* Promoter in Lung Epithelial Cells

Table S1.

Primers for amplification of IL33 promoter, point mutagenesis in IL33 promoter	
Prom fw	GCGAAGCTTACCATTGAGTACAACCAGAA
Prom rev	AATCCATGGTATTCAGTCTTACCTTGTGA
SNP replace Promoter fw	TTTAATAGTTACGAGAGCAT
SNP replace Promoter rev	ATGCTCTCGTAACTATTAAA
CREB1 site mutation A fw	TTTAATAGCCCCAAGAGCAT
CREB1 site mutation A rev	ATGCTCTTGGGGCTATTAAA
CREB1 site mutation G fw	TTTAATAGCCCCGAGAGCAT
CREB1 site mutation G rev	ATGCTCTCGGGGCTATTAAA
Primers for amplification of fragments for pull-down assay	
Pull-down fw	GAAAGGCAGATCAGGAGAGA
Pull-down rev	TTCATTCCCACAACACCGAT
Pul-down control fw	TGATCTCGAACTCCTGACCT
Pull-down control rev	GTGCTGTGATTACAGGTGTG
Primers for real-time RT-PCR	
CREB1 fw	ACCACCGGTAACTAAATGACCA
CREB1 rev	CCCATTGGGCAGCTGTACT
β-actin fw	TGCGTGACATTAAGGAGAAG
β-actin rev	GTCAGGCAGCTCGTAGCTCT
siRNAs for CREB1 knockdown	
siRNA sense	GGAGGAGAGUCGCUACCUAtt
siRNA antisense	UAGGUAGCGACUCUCCUCCtg
Scrambbled sense	GGUCGGCACACGUGAUGAAtt
Scrambbled antisense	UUCAUCACGUGUGCCGACCtg

Fw: forward primer; rev: reverse primer.
